# Persistent mitochondrial and endothelial dysfunction in non-hospitalized patients with Long COVID

**DOI:** 10.3389/fmed.2026.1921822

**Published:** 2026-08-20

**Authors:** Rie Ono, Shin Takayama, Michiaki Abe, Ryutaro Arita, Koh Onodera, Kota Ishizawa, Takeshi Kanno, Natsumi Saito, Akiko Kikuchi, Takaaki Abe, Tadashi Ishii

**Affiliations:** 1 Department of Kampo and Integrative Medicine, Tohoku University Graduate School of Medicine, Sendai, Miyagi, Japan; 2 Department of Kampo Medicine, Tohoku University Hospital, Sendai, Miyagi, Japan; 3 Department of Anesthesiology and Perioperative Medicine, Tohoku University Hospital, Sendai, Miyagi, Japan; 4 Department of Education and Support for Regional Medicine, Tohoku University Hospital, Sendai, Miyagi, Japan; 5 Division of Gastroenterology, Tohoku University Graduate School of Medicine, Sendai, Miyagi, Japan; 6 R&D Division of Career Education for Medical Professionals, Medical Education Center, Jichi Medical University, Shimotsuke, Tochigi, Japan; 7 Department of Clinical Biology and Hormonal Regulation, Tohoku University Graduate School of Medicine, Sendai, Miyagi, Japan; 8 Division of Nephrology, Endocrinology, and Vascular Medicine, Tohoku University Graduate School of Medicine, Sendai, Japan

**Keywords:** biomarkers, endothelial dysfunction, growth differentiation factor 15 (GDF-15), Long COVID, mitochondrial dysfunction, vascular cell adhesion molecule-1 (VCAM-1)

## Abstract

Long COVID, a major post-acute infection syndrome (PAIS), characterized by prolonged or new-onset symptoms following acute COVID-19, critically affects patients' quality of life. Establishing easily measurable and objective biomarkers that reflect disease severity is essential for clinical management. This retrospective cohort study evaluated serum levels of growth differentiation factor 15 (GDF-15), a mitochondrial stress marker, and vascular cell adhesion molecule-1 (VCAM-1), a vascular endothelial stress marker, in 32 patients with Long COVID who presented with persistent systemic or neurological symptoms and had not required acute-phase hospitalization. Serum GDF-15, VCAM-1, and inflammatory cytokine levels were measured at the initial visit and at 3 and 6 months. Differences between patients who recovered at 6 months and those who did not (non-improved group) were analyzed using linear mixed-effects models (LMMs). Most inflammatory cytokines remained near their lower detection limits and were excluded from the longitudinal analysis. The LMMs revealed sustained elevation of both GDF-15 (*p* = 0.02) and VCAM-1 (*p* = 0.03) levels in the non-improved group. These findings suggest that longitudinal tracking of these two markers may offer clinically relevant insights into disease activity and treatment-resistant pathophysiology in mild acute-phase Long COVID.

## 1 Introduction

Coronavirus disease 2019 (COVID-19), caused by severe acute respiratory syndrome coronavirus 2 (SARS-CoV-2), has resulted in a pandemic with more than 770 million patients and 7 million deaths (1). Although the initial clinical focus was primarily on patients with acute severe illness, the persistence or onset of new symptoms following

the acute phase, recognized as a major post-acute infection syndrome (PAIS) and termed the “post-COVID-19 condition” (commonly referred to as Long COVID), has emerged as a secondary major challenge. An estimated 6%−20% of individuals develop Long COVID following COVID-19 (2, 3), leading to decreased quality of life (QOL) and work productivity (4, 5). Furthermore, approximately 20% of affected individuals reportedly experience limitations in their activities of daily living (6, 7). Notably, Long COVID can develop even in patients who experience mild acute disease and do not require hospitalization (8, 9). Because the majority of individuals with SARS-CoV-2 infection do not require hospitalization, a substantial number of individuals are potentially affected, resulting in a profound socioeconomic impact. Moreover, as the prolonged clinical course of Long COVID has been well documented (10, 11), a large number of people experience persistent symptoms, making management of Long COVID a clinical challenge.

Given the high incidence of Long COVID and its widespread public recognition, which is amplified by social media, patients presenting with symptoms suggestive of Long COVID are common in routine clinical practice. However, owing to the lack of established biomarkers, clinical diagnosis and evaluation of response to therapy currently rely largely on patient-reported symptoms, which complicates both clinical management and the development of new therapies (12). Consequently, identifying accessible and actionable biomarkers as objective clinical indices remains a public health priority.

Multiple studies have been conducted to address this need. These studies have provided valuable insights into the underlying biological perturbations associated with Long COVID (13–17) and have highlighted the utility of specific inflammatory markers and indicators of immune dysregulation. Nevertheless, many of these studies are limited by the heterogeneity of their study populations, which often include patients with varying degrees of acute disease severity, ranging from non-hospitalized individuals to those requiring intensive care. Furthermore, the majority of previous studies have had cross-sectional designs, assessing the disease at a single time point. To rigorously eliminate the confounding effects of acute severe organ damage or intensive care interventions, we restricted the study population to patients who were not hospitalized during the acute phase and investigated biomarker dynamics longitudinally.

In our previous analysis of patients with COVID-19 who were not hospitalized during the acute phase of infection, we demonstrated that growth differentiation factor 15 (GDF-15), a marker of mitochondrial stress (18), may serve as a predictive biomarker for the development of Long COVID (19). Furthermore, our previous study revealed a pronounced increase in vascular cell adhesion molecule-1 (VCAM-1), a marker of endothelial dysfunction (20), without a concomitant increase in the levels of systemic inflammatory markers. Mitochondria function not only in energy production and maintaining cellular homeostasis but also in the host response to viral infection (21). However, an excessive response to infection may lead to mitochondrial dysfunction and trigger abnormal immune responses (22, 23). Moreover, the vascular endothelium, which is a major target of SARS-CoV-2 (24), is damaged by immune dysregulation (25). Thus, endothelial and mitochondrial dysfunction may form a mutually perpetuating vicious cycle (26). Therefore, we hypothesized that mitochondrial dysfunction and endothelial damage play central roles in driving persistent fatigue and neurocognitive impairment, and that markers of these dysfunctions could serve as biomarkers for Long COVID.

The objective of this study was to measure GDF-15 and VCAM-1 longitudinally in patients with Long COVID who did not require hospitalization during the acute phase of COVID-19, and to evaluate their potential as biomarkers for Long COVID.

## 2 Materials and methods

### 2.1 Ethics

The study was conducted in accordance with the Declaration of Helsinki and the Tokyo Guidelines for Human Research. It was approved by the Ethics Committee of Tohoku University Graduate School of Medicine (approval number: 2022-1-801). As this study had a retrospective study design and used residual samples, the requirement for individual informed consent was waived by the Ethics Committee. Instead, an opt-out method was employed, and information regarding the study was publicly disclosed, providing patients or their proxies with the opportunity to refuse the use of their samples and data for research. With respect to sample storage, written informed consent for preserving blood samples in the biobank was obtained from the patients at their initial visit. This biobanking procedure was approved by the Ethics Committee of Tohoku University Graduate School of Medicine as a research project of the Biobank Division at the Center for Personalized Medicine (approval number: 2017-1-346). This manuscript was prepared in accordance with the Strengthening the Reporting of Observational Studies in Epidemiology (STROBE) guidelines.

### 2.2 Study design and participants

This single-center, retrospective, longitudinal cohort study was conducted at the Department of General Medicine at Tohoku University Hospital. In accordance with the National Institute for Health and Care Excellence (NICE) guidelines, Long COVID was defined as symptoms persisting for more than 4 weeks after the onset of COVID-19 (27). We enrolled non-hospitalized patients who met these criteria and visited our department between January 2022 and August 2024 during the acute phase of SARS-CoV-2 infection. However, during the pandemic, a severe shortage of hospital beds necessitated providing oxygen therapy to some patients in temporary isolation facilities. Because the clinical severity of individuals who received oxygen therapy at temporary isolation facilities was equivalent to that of hospitalized patients, they were excluded from the study.

The analysis was restricted to patients who were followed up for at least 3 months and had paired banked serum samples available at both the initial visit and the 3-month follow-up visit. For patients with a 6-month follow-up visit, serum samples obtained at the 6-month follow-up visit were also included in the analysis, if available.

To evaluate the systemic pathophysiology, patients presenting with fatigue, neurocognitive impairment (e.g., brain fog), or myalgia/arthralgia were included. In contrast, those with exclusively localized symptoms (e.g., taste or smell disorders, cough) were excluded. The additional exclusion criteria were: (1) unconfirmed SARS-CoV-2 infection (without positive polymerase chain reaction or antigen test results); (2) age under 18 years or over 65 years; (3) initial visit >180 days post-onset; (4) pre-existing comorbidities providing alternative diagnoses for the symptoms; and (5) pregnancy.

Two patients who were enrolled in our previously published study on acute-phase biomarkers (19) subsequently developed prolonged symptoms and were also included in this analysis.

### 2.3 Definition of the clinical outcome

During the follow-up period, all patients received pharmacological treatment under standard health insurance coverage to alleviate their symptoms. Patients were classified into two groups based on their clinical outcome 6 months after the initial visit: the recovered group (those whose symptoms resolved), and the non-improved group (those with persistent symptoms). Patients who showed clinical improvement and were discharged from outpatient care prior to the 6-month follow-up visit were included in the recovered group.

“Clinical recovery” was defined as the resumption of pre-COVID-19 social and daily activities, including returning to work or school, as documented in medical records, without using standardized criteria derived from symptom questionnaires. This functional outcome was prioritized because, from a clinical perspective, subjective metrics such as the Visual Analog Scale (VAS) exhibit significant interindividual variability and frequently dissociate from actual social functioning.

To prevent bias, clinical outcomes were definitively categorized prior to the collective measurement of biomarkers.

### 2.4 Sample collection and biomarker measurement

Venous blood samples were transferred immediately after collection to the Tohoku University Medical Megabank Organization, where serum separation via centrifugation (at 1,700 × *g* for 10 min at 4 °C) and immediate storage at −80 °C were performed by the biobank staff.

For biomarker analysis, stored serum samples were retrieved from the biobank and transported under frozen conditions to Filgen, Inc. (Nagoya, Japan). In addition to GDF-15 and VCAM-1, systemic inflammatory markers—including interleukin (IL)-6, tumor necrosis factor-alpha (TNF-α), and IL-10—were also measured, given that previous studies have suggested an association between these inflammatory cytokines and the pathophysiology of Long COVID.

Biomarker levels were quantified using enzyme-linked immunosorbent assay (ELISA) kits according to the manufacturers' instructions: GDF-15 was measured using the MILLIPLEX^®^ Human Cancer Metastasis Biomarker Magnetic Bead Panel (Merck, Darmstadt, Germany), whereas VCAM-1, IL-6, TNF-α, and IL-10 were measured using the Luminex^®^ Discovery Assay Human Premixed Multi-Analyte Kit (R&D Systems, Minneapolis, MN, USA). The detection ranges of the assays were 2 to 10,000 pg/ml for GDF-15, 8.85 to 2,150 ng/ml for VCAM-1, 4.53 to 1,100 pg/ml for IL-6, 8.23 to 1,000 pg/ml for TNF-α, and 4.12 to 1,000 pg/ml for IL-10. For values outside these ranges, concentrations were extrapolated using the regression equation of the standard curve. In cases in which the value could not be calculated using the regression equation, the result was treated as zero.

### 2.5 Statistical analysis

The normality of the data distribution was evaluated using the Shapiro–Wilk test. Because some variables did not have a normal distribution, non-parametric methods were applied. Continuous variables were expressed as medians with IQRs, and comparisons between the recovered and non-improved groups were performed using the Mann–Whitney *U*-test. Categorical variables were reported as frequencies and percentages, and groups were compared using the chi-square test or Fisher's exact test (when *N* ≤ 4).

To evaluate longitudinal differences in biomarkers between groups, we applied LMMs with restricted maximum likelihood (REML) estimation, including a random intercept for each patient to account for repeated measures. This approach allowed us to include patients with missing data at the 6-month follow-up without relying on listwise deletion, thereby preserving statistical power despite the limited sample size. Given the number of patients, the model was parsimoniously constructed using three fixed-effect parameters to avoid overfitting: the overall group difference (group), the time effect (time), and their interaction (group × time). The statistical significance of the longitudinal trajectories was evaluated using *F*-statistics and *p*-values derived from this model, while the data in the figures were plotted using the means and standard errors of the raw data.

No formal *a priori* sample size calculation was performed. Rather, we included all consecutive patients who met the eligibility criteria and had paired serum samples available during the study period.

Statistical analyses were performed using GraphPad Prism version 10.6.1 (GraphPad Software, Boston, MA, USA). Statistical significance was set at a two-sided *p* value < 0.05.

## 3 Results

### 3.1 Baseline characteristics

**Figure 1:**
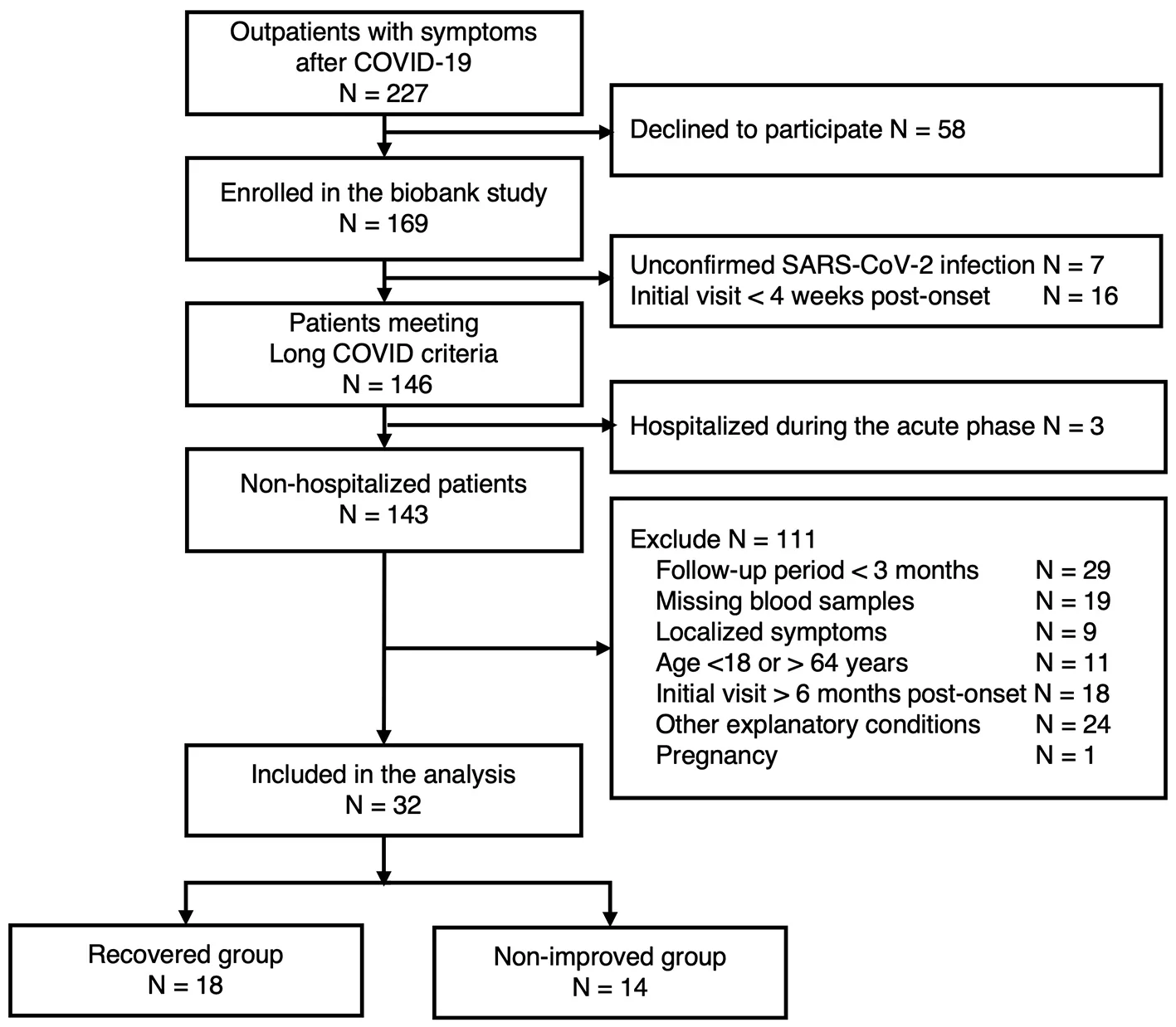
Study flowchart detailing the patient enrollment process. A total of 227 individuals were initially assessed for eligibility. Following the application of the exclusion criteria, 32 patients were enrolled and divided into recovered (*n* = 18) and non-improved (*n* = 14) groups for longitudinal biomarker analysis.

Among the patients who visited our department during the study period, 32 met the inclusion criteria and were included in this study (Figure 1). Among the 32 participants, 14 (44%) were female, and the median age was 37 years [interquartile range (IQR), 30–49]. Based on their 6-month clinical outcomes, 18 patients were assigned to the recovered group and 14 to the non-improved group.

Table 1 summarizes the baseline demographic characteristics, presenting symptoms, and comprehensive quality of life (QOL) scores—assessed using the EuroQol 5-Dimensions 5-Levels (EQ-5D-5L) and Short Form-12 Health Survey (SF-12)—according to the group. Age, sex, body mass index, medical history, and the median interval from COVID-19 onset to the initial outpatient visit did not differ significantly between the groups.

**Table 1:** Baseline clinical characteristics of patients with Long COVID, stratified by recovery status.

Variable	All	Non-improved	Recovered	*p*
	(*N* = 32)	(*N* = 14)	(*N* = 18)	
Age (years), median (IQR)	37 (30-49)	36 (27-55)	37 (32-45)	0.96
Female sex, *n* (%)	14 (44)	7 (50)	7 (39)	0.72
Body mass index (kg/m^2^) median (IQR)	23.2 (20.0–26.5)	21.0 (19.5–28.8)	24.1 (21.1–26.5)	0.39
Smoking, *n* (%)	7 (23)	2 (14)	5 (31)	0.41
Alcohol, *n* (%)	12 (40)	4 (29)	8 (50)	0.22
Time from COVID-19 onset to first outpatient visit (days), median (IQR)	62 (46–118)	84 (45–120)	57 (45–124)	0.37
Comorbidities			
Number of comorbidities per patient, median (IQR)	2 (1-3)	2 (1-2)	2 (1-3)	0.94
Respiratory, *n* (%)	12 (38)	5 (36)	7 (39)	>0.99
Digestive, *n* (%)	10 (31)	5 (36)	4 (28)	0.71
Orthopedic, *n* (%)	5 (16)	3 (21)	2 (11)	0.63
Genital, *n* (%)	5 (16)	1 (7)	4 (28)	0.35
Psychological, *n* (%)	5 (16)	3 (21)	2 (11)	0.63
Cardiovascular, *n* (%)	5 (16)	4 (27)	1 (6)	0.14
Dyslipidemia, *n* (%)	4 (13)	2 (14)	2 (11)	>0.99
Neurology, *n* (%)	3 (9)	1 (7)	2 (11)	>0.99
Diabetes mellitus, *n* (%)	2 (6)	1 (7)	1 (6)	>0.99
Immunology/allergy, *n* (%)	1 (3)	1 (7)	0 (0)	0.44
Symptoms			
Fatigue, *n* (%)	29 (91)	13 (93)	16 (89)	>0.99
Headache, *n* (%)	19 (59)	9 (64)	10 (56)	0.62
Short of breath, *n* (%)	17 (53)	9 (64)	8 (44)	0.26
Brain fog, *n* (%)	15 (47)	9 (64)	6 (33)	0.08
Musculoskeletal pain, *n* (%)	12 (38)	5 (36)	7 (39)	0.85
Sputum, *n* (%)	7 (22)	4 (29)	3 (17)	0.67
Cough, *n* (%)	6 (19)	5 (36)	1 (6)	0.06
Olfactory dysfunction, *n* (%)	5 (16)	1 (7)	4 (22)	0.35
Taste dysfunction, *n* (%)	3 (9)	1 (7)	2 (11)	> 0.99
Number of symptoms per patient, median (IQR)	4 (3-5)	5 (3-6)	3 (2-5)	0.14
Comprehensive QOL			
EQ-5D-5L, HR-QOL, median (IQR)	0.695 (0.574–0.779)	0.599 (0.489–0.771)	0.742 (0.649–0.794)	0.11
EQ VAS, median (IQR)	40 (34-60)	40 (30-50)	40 (35-65)	0.17
SF-12				
PCS, median (IQR)	34.3 (27.9–44.3)	30.0 (22.6–45.8)	40.5 (32.4–45.9)	0.11
MCS, median (IQR)	42.8 (39.3–46.2)	42.4 (40.1–44.8)	44.7 (39.5–49.6)	0.59
RCS, median (IQR)	38.0 (28.8–46.9)	34.6 (26.1–41.9)	41.4 (28.9–49.2)	0.28

IQR, interquartile range; QOL, quality of life; EQ-5D-5L, EuroQol 5-dimensions 5-levels; EQ VAS, EuroQol Visual Analog Scale; SF-12, short form-12 health survey: PCS, physical component summary: MCS, mental component summary: RCS, role/social component summary.

P-values were calculated using the Mann–Whitney U-test for continuous variables and Fisher's exact test for categorical variables. Only major or frequently reported symptoms and comorbidities are presented.

Patients typically presented with multiple concurrent symptoms (median 4; IQR, 3–5), with fatigue being the most prevalent (29 patients, 91%). Although the prevalence of specific symptoms and baseline QOL scores did not differ significantly between the two groups, brain fog (64 vs. 33%, *p* = 0.08) and cough (36 vs. 6%, *p* = 0.06) were more common in the non-improved group.

### 3.2 Biomarker measurements

A total of 81 serum samples were measured. In the non-improved group, samples were collected at the initial visit (*n* = 14), 3-month follow-up (*n* = 14), and 6-month follow-up (*n* = 7). In the recovered group, samples were collected at the initial visit (*n* = 18), 3-month follow-up (*n* = 18), and 6-month follow-up (*n* = 10). The biomarker values are summarized in Table 2.

**Table 2:** Summary of longitudinal biomarker levels in the recovered and non-improved groups.

Time point/ Biomarker	Non-improved	Recovered	*p*
Initial visit			
GDF-15 (pg/ml)	425 (378–653)	445 (365–563)	0.7
VCAM-1 (ng/ml)	860 (624–978)	678 (548–820)	0.18
IL-6 (pg/ml)	0.27 (0–0.82)	0.20 (0–0.43)	0.34
TNF-α (pg/ml)	2.9 (2.2–4.4)	3.0 (2.4–3.4)	0.89
IL-10 (pg/ml)	0 (0–0.05)	0 (0–0.67)	0.6
3 months			
GDF-15 (pg/ml)	520 (335–755)	445 (330–523)	0.28
VCAM-1 (ng/ml)	859 (559–941)	636 (489–827)	0.22
IL-6 (pg/ml)	0.35 (0.14–0.89)	0.42 (0–0.76)	0.74
TNF-α (pg/ml)	3.0 (2.1–4.2)	3.2 (2.5–3.9)	0.84
IL-10 (pg/ml)	0 (0–0.13)	0 (0–7.0)	0.2
6 months			
GDF-15 (pg/ml)	680 (340–810)	410 (320–515)	0.07
VCAM-1 (ng/ml)	1,043 (785–1,089)	663 (570–697)	0.004
IL-6 (pg/ml)	0 (0–0.94)	0.58 (0.24–1.4)	0.25
TNF-α (pg/ml)	4.2 (2.6–4.3)	3.8 (3.4–4.8)	0.68
IL-10 (pg/ml)	0 (0–0.19)	0 (0–0.0)	0.55

Data are presented as the median (interquartile range [IQR]). *P* values represent cross-sectional comparisons between the recovered and non-improved groups at each time-point and were calculated using the Mann–Whitney U-test.

GDF-15, growth differentiation factor 15; IL, interleukin; TNF-α, tumor necrosis factor-alpha; VCAM-1, vascular cell adhesion molecule-1.

In terms of the levels of inflammatory cytokines (IL-6, TNF-α, and IL-10), the measured levels were pre-dominantly near the lower limit of the standard curve at all time-points. Specifically, 26 samples (36%) for IL-6 and 59 samples (82%) for IL-10 exhibited fluorescence intensities below the limit of detection. Consequently, these markers were deemed unsuitable for evaluation of their association with Long COVID and were excluded from further longitudinal statistical analyses.

### 3.3 Longitudinal analysis of GDF-15 trajectories

**Figure 2:**
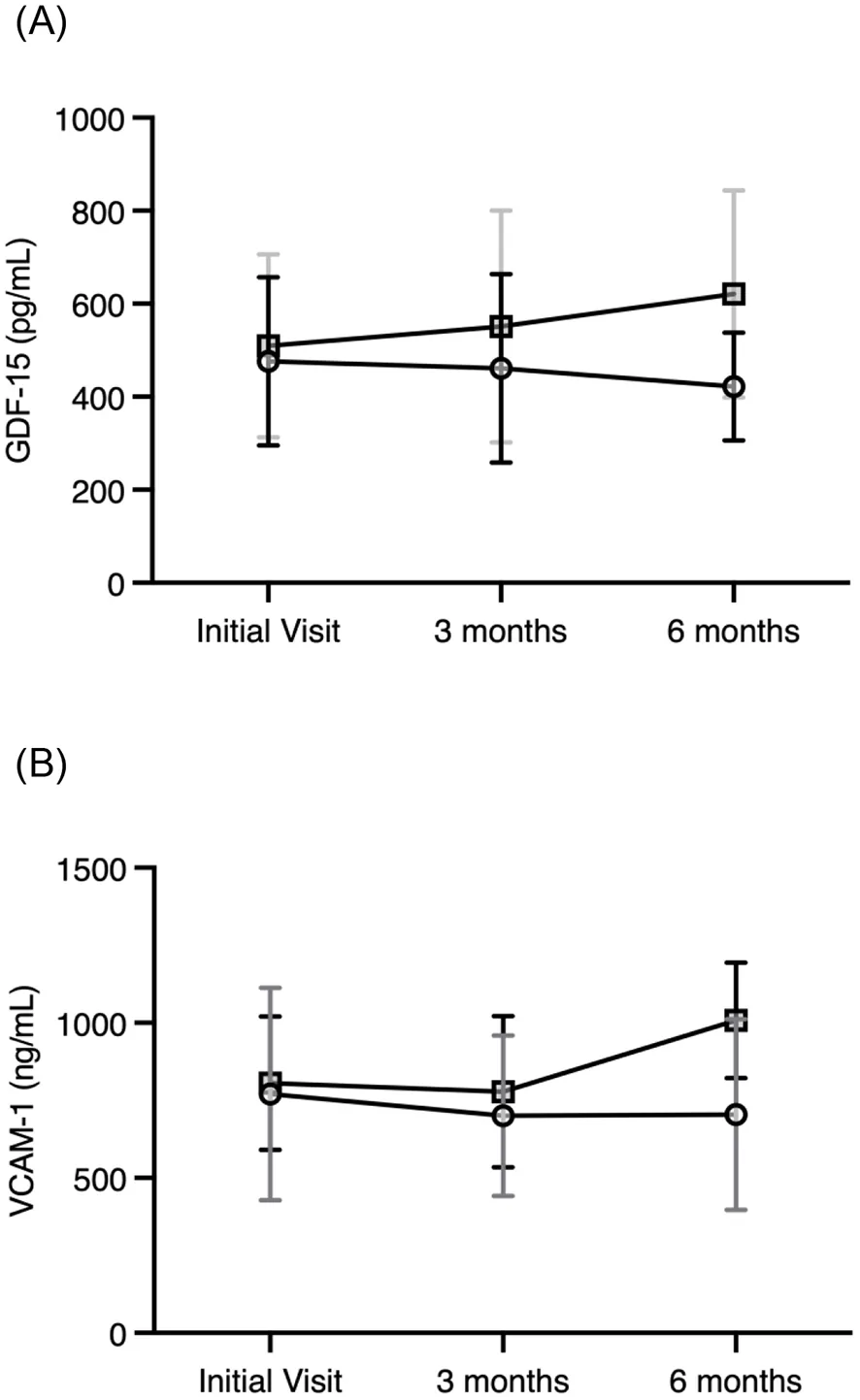
Longitudinal trajectories of serum GDF-15 and VCAM-1 levels in patients with Long COVID. **(A)** Trajectories of serum GDF-15 and **(B)** VCAM-1 levels in the recovered (open circles) and non-improved (open squares) groups. The data are presented as the means ± standard errors of the means (SEMs) of the raw data. Data were analyzed using a linear mixed-effects model (LMM). Sustained elevations of both GDF-15 (*p* = 0.02) and VCAM-1 (*p* = 0.03) were observed in the non-improved group.

We compared serum GDF-15 levels between the non-improved and recovered groups at each time point. Although no significant differences were observed at the initial visit (median: 425 vs. 445 pg/ml, *p* = 0.70) or at 3 months (median: 520 vs. 445 pg/ml, *p* = 0.28), the levels were higher in the non-improved group, with the difference approaching statistical significance at 6 months (median: 680 vs. 410 pg/ml, *p* = 0.07; Table 2). The longitudinal GDF-15 trajectories, evaluated using LMM, are shown in Figure 2A. The LMM analysis revealed a significant main effect of group (*F* = 5.27, *p* = 0.02), indicating that GDF-15 levels were significantly higher in the non-improved group than in the recovered group throughout the observation period (estimated mean difference: 108 pg/ml, 95% confidence interval [CI]: 14 to 201). Conversely, neither the main effect of time (*F* = 0.12, *p* = 0.89) nor the group-by-time interaction (*F* = 0.94, *p* = 0.40) was statistically significant. These results indicate that GDF-15 levels remained relatively stable over time in both groups, and that the non-improved group exhibited a consistently elevated trajectory without significant divergence over time.

### 3.4 Longitudinal analysis of VCAM-1 trajectories

We compared serum VCAM-1 levels between the non-improved and recovered groups at each time point. Although no significant differences were observed at the initial visit (median: 860 vs. 678 ng/ml, *p* = 0.18) or at 3 months (median: 859 vs. 636 ng/ml, *p* = 0.22), the non-improved group exhibited significantly higher levels at 6 months (median: 1,043 vs. 663 ng/ml, *p* = 0.004; Table 2). The longitudinal VCAM-1 trajectories, evaluated using LMM, are shown in Figure 2B. The LMM analysis revealed a significant main effect of group (*F* = 4.68, *p* = 0.03), indicating that the overall VCAM-1 levels were significantly higher in the non-improved group than in the recovered group throughout the observation period (estimated mean difference: 138.7 ng/ml, 95% CI: 11.0 to 266.4). Conversely, neither the main effect of time (*F* = 1.01, *p* = 0.37) nor the group-by-time interaction (*F* = 1.39, *p* = 0.26) was statistically significant. Consistent with the GDF-15 findings, these results demonstrated that VCAM-1 levels remained elevated in the non-improved group across all time points, with no significant temporal variation or divergence in the trajectories over time.

## 4 Discussion

In this study, we investigated biomarkers in Long COVID patients who presented with persistent systemic and neurological symptoms but did not require hospitalization during the acute phase of infection. Although previous studies have highlighted the importance of systemic inflammatory markers (16, 17), such elevations were rarely observed in our cohort. In contrast, we demonstrated that the longitudinal trajectories of GDF-15 and VCAM-1 remained persistently elevated in the non-improved group compared with those in the recovered group (19). This discrepancy with previous findings may be attributed to the frequent inclusion of patients requiring acute-phase hospitalization in earlier study cohorts. Importantly, because all patients in our study received treatment, these biomarker trends might not simply predict the natural prognosis of Long COVID; rather, they may reflect the pathophysiological differences between treatment-resistant and treatment-responsive groups or the effectiveness of the treatment.

GDF-15 is a stress-responsive cytokine that is produced primarily in response to mitochondrial stress and contributes to energy metabolism and immune regulation (18). Our previous study demonstrated that elevated GDF-15 levels during the acute phase of COVID-19 predicted the subsequent development of Long COVID. The current study further revealed a potential association between persistently elevated GDF-15 levels and unfavorable clinical outcomes in patients with Long COVID. Taken together, these findings suggest that underlying mitochondrial dysfunction, as well as a prolonged impairment in mitochondrial homeostasis or repair processes, are involved in the pathophysiology of Long COVID. Previous studies have reported morphological and metabolic abnormalities in the mitochondria of patients with Long COVID (28–30). Furthermore, such mitochondrial dysfunction is associated with the pathophysiology of myalgic encephalomyelitis/chronic fatigue syndrome (ME/CFS) (31) and chronic pain disorders (32). Given the pathophysiological overlap between Long COVID and these conditions, the longitudinal dynamics of GDF-15 observed in our study may closely reflect the core pathophysiology of Long COVID.

VCAM-1 is an adhesion molecule induced by inflammatory stimuli that facilitates leukocyte adhesion and migration (33). In circulation, VCAM-1 exists as a soluble form and serves as a marker of endothelial injury (18). Previous studies have reported that endothelial dysfunction persists even after the acute phase of infection (34). The elevation of VCAM-1 observed in our study likely reflects this ongoing endothelial dysfunction. However, in our previous study, we did not observe an elevation of syndecan-1—a marker reflecting glycocalyx damage—in patients during the acute phase and at 3 months post-onset (19). Therefore, we consider that the persistently elevated trajectory of VCAM-1 observed in the non-improved group suggests a prolonged state of functional endothelial activation and dysfunction, without overt structural damage. Furthermore, previous studies have implicated blood-brain barrier (BBB) dysfunction in the pathogenesis of brain fog. VCAM-1 is involved in BBB disruption and increased permeability (35). Furthermore, reduced cerebral blood flow on single photon emission computed tomography (SPECT) has been reported in patients presenting with brain fog, supporting the hypothesis that underlying endothelial dysfunction may contribute to these neurological symptoms (36). Therefore, the persistent increase in VCAM-1 may closely reflect ongoing vascular pathology extending to the central nervous system. Although earlier studies have reported associations between Long COVID and markers of vascular injury, our study provides novel evidence that VCAM-1 can remain persistently elevated over the long term, even in patients who do not require hospitalization during the acute phase. Notably, the significant difference in VCAM-1 levels observed in the non-recovered and improved groups at 6 months might be partially amplified by the attrition bias inherent to real-world data: patients who achieved early clinical recovery were more likely to discontinue follow-up; therefore, the 6-month data may disproportionately include patients with more persistent symptoms. However, by analyzing the data using a LMM, which accommodates missing data, we mitigated this effect and confirmed a consistently elevated trajectory throughout the observation period.

We also considered the potential relationship between GDF-15 and VCAM-1. Recent large-scale comprehensive profiling studies have reported that persistent immune dysregulation, complement activation, and thromboinflammation serve as fundamental drivers of Long COVID (37–39). Mitochondria play a crucial role in immune regulation, and their dysfunction can induce endothelial damage in a cascading manner. Therefore, the persistent elevations of serum GDF-15 and VCAM-1 observed in our cohort are likely not isolated events but rather reflect intertwined aspects of this complex pathophysiological network, linking mitochondrial dysfunction to endothelial activation. Furthermore, capturing these systemic changes using easily accessible and minimally invasive peripheral blood samples holds potential for future clinical application in the routine management of Long COVID.

This study has several limitations. First, this was a single-center study with a relatively small sample size. Second, some patients were lost to follow-up before the 6-month follow-up visit. To address this, we applied an LMM without the list-wise deletion of missing cases; however, this did not overcome the limited statistical power owing to the small sample size. In addition, an assessment of attrition bias was not feasible owing to the small sample size; therefore, the possibility of attrition bias cannot be excluded.

Third, as this study was conducted within the scope of routine clinical practice, a comparison with healthy controls was not performed. However, because blood levels of GDF-15 and VCAM-1 are influenced by age and comorbidities, there is significant interindividual variability. Therefore, it is challenging to evaluate these markers based solely on their absolute values; rather, as demonstrated in this study, tracking the longitudinal trajectories from each patient's baseline holds significant clinical relevance.

Finally, the therapeutic interventions during the observation period were not standardized, and potential biases arising from differences in symptomatic treatments cannot be excluded.

Considering these limitations, larger multicenter prospective studies that use standardized treatment protocols are warranted to validate the longitudinal biomarker dynamics observed in our cohort. However, given that no definitive treatment for Long COVID has yet been established, restricting patients to a standardized treatment protocol would require careful ethical consideration.

In conclusion, this exploratory study demonstrated that in patients with mild acute-phase COVID-19 who do not require hospitalization, serum GDF-15 and VCAM-1 levels remain persistently elevated in those with Long COVID. These findings suggest a potential underlying link between sustained mitochondrial and endothelial stress and the pathophysiology of persistent symptoms. While these biomarkers cannot establish a direct causal mechanism and are not yet ready for immediate clinical implementation, they represent promising candidates that warrant further validation in larger prospective cohorts.

## Data Availability

The datasets generated and/or analyzed during the current study are not publicly available owing to privacy and ethical restrictions regarding patient data but are available from the corresponding author on reasonable request.
